# Effectiveness of breathing exercise on the duration of labour: A systematic review and meta-analysis

**DOI:** 10.7189/jogh.13.04023

**Published:** 2023-03-10

**Authors:** Alwin Issac, Shalini Ganesh Nayak, Priyadarshini T, Deepthy Balakrishnan, Kurvatteppa Halemani, Prabhakar Mishra, Indumathi P, Vijay VR, Jaison Jacob, Shine Stephen

**Affiliations:** 1 All India Institute of Medical Sciences, Bhubaneswar, India; 2 All India Institute of Medical Sciences, Gorakhpur, India; 3 Sanjay Gandhi Postgraduate Institute of Medical Sciences, Lucknow, India

## Abstract

**Background:**

Prolonged labour intensifies labour pain, and failure to address labour pain may lead to abnormal labour and augments the usage of operative interventions. Prolonged labour is common among women, resulting in maternal morbidity, increased caesarean section (CS) rates, and postpartum complications. It may bring forth negative birth experiences that may increase the preference for CS. There is a dearth of evidence concerning the effectiveness of breathing exercises on the duration of labor. As per our knowledge, this is the first systematic review and meta-analysis on the effect of breathing exercises on the duration of labor. This systematic review and meta-analysis aimed to appraise the evidence concerning the effectiveness of breathing exercises on the duration of labour.

**Methods:**

Electronic databases MEDLINE, Cumulative Index to Nursing and Allied Health Literature (CINAHL), EMBASE, Web of Science, SCOPUS, and ClinicalKey were searched for randomized controlled trials, quasi-experimental studies published in the English language between January 2005 to March 2022 that reported on the effectiveness of breathing exercises on the duration of labour. Duration of labour was the primary analysed outcome. The secondary outcomes assessed were anxiety, duration of pain, APGAR scores, episiotomy, and mode of delivery. Meta-analysis was done using RevMan v5.3.

**Results:**

The reviewed trials involved 1418 participants, and the study participants ranged from 70 to 320. The mean gestational weeks of the participants among the reported trials was 38.9 weeks. Breathing exercise shortened the duration of the intervention group’s second stage of labour compared with the control group.

**Conclusions:**

Breathing exercise is a beneficial preventive intervention in shortening the duration of second stage of labour.

**Registration:**

The review protocol was registered with PROSPERO (CRD42021247126).

Prolonged labour (PL) or dystocia is one of the most common birth complications and the most common indication for instrumental delivery or delivery by emergency caesarean section (CS) [1]. Globally, PL is prevalent among 8% of women giving birth [2]. Women with PL bring forth a negative birth experience, a risk factor for a later wish for a CS [3]. The global increase in the CS rate is accompanied by numerous maternal morbidities [4,5]. Improved maternal health is one of the United Nations Millennium Development Goals [6]. According to the World Health Organization (WHO), CS rates higher than 10% at a population level are associated with increased maternal and neonatal mortality rates [2]. The process of labour and childbirth brings forth numerous physical and psychological demands resulting in maternal stress with the release of the hormone cortisol. Heightened stress and release of cortisol hormone have a detrimental effect on childbirth, lactation, and infant-mother bonding [7].

PL intensifies labour pain. Failure to address labour pain may lead to abnormal labour and augments the usage of operative interventions. The labour pain associated with cervical dilatation and uterine contractions progressively increases in severity. Epidural analgesia (EA) prolongs the second stage of labour, though it is the gold standard and most effective treatment for labour pain [8]. EA is an independent risk factor for CS, instrumental delivery, and abnormal foetal head position at delivery [9]. Early use of oxytocin and amniotomy is performed to accelerate the slow progress of labour and encourage cervical dilation [10,11]. However, oxytocin augmentation is associated with emergency CS, uterine hyperstimulation, and low APGAR scores in newborns, which can affect women’s and newborn’s health [12-14]. PL is associated with a lower APGAR score at one minute, increased postpartum haemorrhage, increased incidence of asphyxia, and adverse birth experience [3,15,16].

WHO has defined complementary and alternative medicine (CAM) as a “broad set of health care practices that are not part of that country’s tradition or conventional medicine and are not fully integrated into the dominant healthcare system” [2]. CAM is categorised into the alternative medical system, mind-body interventions, biologically based treatment, energy therapies, and manipulative and body-based methods. Increasing evidence on the effectiveness of CAM has significantly accentuated its utilization among pregnant women and postpartum mothers [17,18]. CAM is effective in reducing labour pain [19], pregnancy-related back and pelvic pain [20], nausea and vomiting during pregnancy [21], EA requirement [22], augmenting normal vaginal birth [23], and postpartum uterine after-pain [24].

Breathing exercise is regarded as the most practised, effective, and expected CAM therapy in future [25]. Breathing exercises stimulate the parasympathetic nervous system, leading to increased blood oxygenation, thereby releasing endorphins, which would decrease the heart rate and bring forth a sense of calmness. Simultaneously, endorphins suppress the sympathetic nervous system resulting in decreased release of the stress hormone cortisol [26]. Nurses are uniquely positioned to administer breathing exercises to improve maternal and neonatal outcomes and reduce elective CS rates.

There is a dearth of evidence concerning the effectiveness of breathing exercises on the duration of labour. As per our knowledge, this is the first systematic review and meta-analysis on the effect of breathing exercises on the duration of labour. The findings of this review would provide new insight into the effectiveness of breathing exercises in the scientific community. With this intention, we conducted a systematic review and meta-analysis of the RCTs that assessed the effectiveness of breathing exercises on the duration of labour.

## METHODS

This systematic review and meta-analysis aimed to appraise the evidence concerning the effectiveness of breathing exercises on the duration of labour. The review protocol was registered with PROSPERO (CRD42021247126). The guidelines of Cochrane collaboration were adopted to carry out this systematic review and meta-analysis [27] and reported using the Preferred Reporting Items for Systematic Reviews and Meta-analysis (PRISMA) statement [28]. A comprehensive search strategy was developed using keywords or key terms connected to (population or participant, intervention, comparator or control, and outcomes (PICO) in MEDLINE and tailored into different databases. The population involved women in the first or second stage of labour who had a random allocation to either the experimental group that received breathing exercises as the intervention or a control group that received the usual or routine care of the hospital. Those receiving breathing exercises in combination with other therapies such as aroma therapy, reflexology, massage etc. were excluded. Duration of labour was the primary outcome analysed in this systematic review and meta-analysis. The secondary maternal and foetal outcomes assessed were anxiety, pain, APGAR scores, and mode of delivery.

The following combinations of MeSH (Medical Subject Heading) terms or keywords were used: labour, obstetrics, parturition, delivery, breathing exercises, exercise, breathing technique, breathing practices, diaphragmatic breathing, respiration, women, gravidity, pregnant women, labour stage first, and labour stage second. Two authors (AI, SGN) independently searched the electronic databases MEDLINE, CINAHL, EMBASE, Web of Science, SCOPUS, and ClinicalKey for randomized controlled trials (RCTs), quasi-experimental studies published in the English language between January 2005 and March 2022 that reported on the effectiveness of breathing exercises on the duration of labour. The identified articles were imported into Rayyan software [29]. The duplicate records were identified and excluded. The references of the relevant articles that fulfilled the inclusion criteria were manually searched for additional studies. All the remaining original full-text articles were screened in accordance with the inclusion criteria.

**Figure 1:**
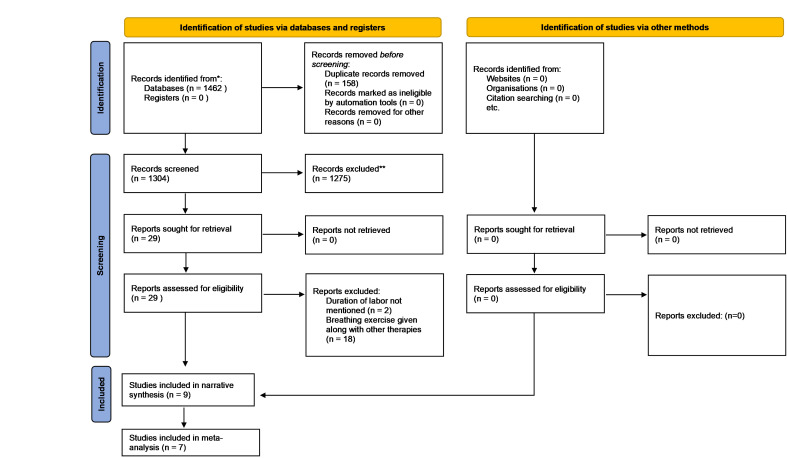
Flow diagram of study selection.

The search strategy identified 1462 articles, and 158 duplicate records were excluded. The title and abstract of the remaining articles were assessed, and 1275 articles were removed as they did not match the inclusion criteria of the review in accordance with PICO. The full text of the remaining 29 articles was assessed, and 20 articles were removed as they did not match the inclusion criteria of the review. The reasons for excluding the articles were that the study involved breathing exercises combined with other therapies as an intervention, and the duration of labour was not measured as an outcome. The remaining nine articles were included in the qualitative and narrative synthesis. Another trial evaluated the effectiveness of breathing exercises on the duration of the second stage of labour. However, this trial was not involved in the meta-analysis as the duration of labour was not reported in seconds, minutes, or hours. Instead, the trial reported the number of participants who had a prolonged second stage of labour in the intervention and control group [30]. Hence, six trials were included in the meta-analysis that assessed the effectiveness of breathing exercises on the duration of labour. The flow diagram of the study selection process is depicted in **Figure 1**.

**Figure 2:**
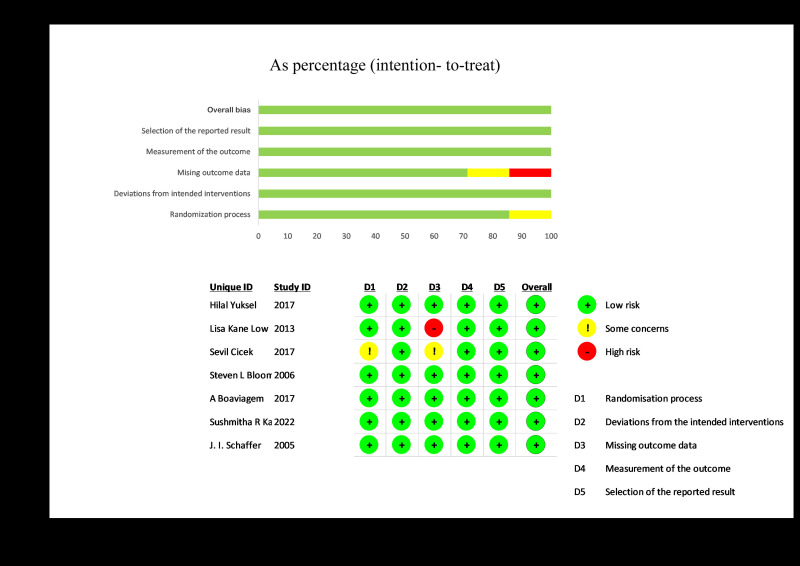
Risk of bias graph.

The Cochrane risk of bias tool-2 (RoB-2) [31] was used to assess the quality of the included RCTs (**Figure 2**), and the ROBINS-1 [32] tool was used to evaluate the quality of the quasi-experimental studies. The RoB-2 tool assess the RCTs under five domains namely “bias arising from the randomization process”, “bias due to deviations from intended interventions”, “bias due to missing outcome data”, “bias in measurement of the outcome”, and “bias in selection of the reported result”. The signalling questions in each domain of the tool aim to elicit information relevant to an assessment of risk of bias. Responses to the signalling questions feed into an algorithm to guide users of the tool to judge about the risk of bias. The response options for the signalling questions are “yes”, “probably yes”, “probably no”, “no”, and “no information”. Responses of “yes” and “probably yes” have the same implication for risk of bias, as do responses of “no” and “probably no”. The “no information” response to be used only when both (i) insufficient details are reported to permit a response of “probably yes” or “probably no”, and (ii) in the absence of these details it would be unreasonable to respond “probably yes” or “probably no” in the circumstances of the trial. The responses to the signalling questions would result into a risk of bias judgment as “low risk of bias”, “some concerns”, and “high risk of bias”.

The ROBINS-1 tool assess the non-randomized studies under seven domains namely “bias due to confounding”, “bias in selection of participants into the study”, “bias in classification of interventions”, “bias due to deviations from intended interventions”, “bias due to missing data”, “bias in measurement of outcomes”, and “bias in selection of the reported result”. The interpretation of each domain and overall risk of bias judgments in ROBINS-1 tool result into “low risk of bias”, “moderate risk of bias”, “serious risk of bias”, “critical risk of bias”, and “no information”.

A data extraction sheet was developed to extract data from the included studies. Data was extracted independently by two reviewers (AI, SGN) and was reviewed by third reviewer (PT). The items of data extraction form were author (s), year of publication, country, the aim of the study, study design, sample size, participant characteristics, intervention characteristics, characteristics of the control group, and key findings reported by the author(s).

The primary outcome measure (duration of labour) was compared between participants who received breathing exercise intervention and the participants in the control group who received routine/usual care in the hospital in each study. The meta-analysis was performed to pool the results of the RCTs. The weighted score of the studies was calculated. The effect size for breathing exercise was estimated for the continuous outcome (duration of labour) in terms of pooling mean (95% confidence interval (CI)) from the standardized mean difference (SMD) with 95% CI of the individual studies. Similarly, when data were recorded in number and percentage, the risk ratio or relative risk (RR) was calculated for the intervention group compared to the control group. All the results were incorporated with a corresponding 95% CI. The heterogeneity of the included trials was analysed using the *I*^2^ value with a random effect model. The data were analysed and pooled using the software RevMan v5.3.

## RESULTS

The assessment of the quality of the included RCTs were done with the ROB-2 tool. All the RCTs were judged to have “low risk of bias” in domain of “bias due to deviations from intended interventions”, “bias in measurement of the outcome”, and “bias in selection of the reported result”. The article by Lisa Kane Low [33] was judged to have “high risk of bias” in domain of “bias due to missing outcome data”. 

The included articles involved 1418 participants, and the size of the study participants ranged from 70 to 320. The mean age of the participants was 23.6 years. The mean gestational weeks of the participants among the five reported trials was 38.9 weeks, while two trials did not report the gestational weeks of the study participants [33,34]. The summary data of the included studies are presented in **Table 1**.

**Table 1:** Summary of data from all included studies

Author, year, country	Aim of the study, study design, sample size	Participant characteristics	Intervention characteristics	Control group characteristics	Key findings reported by author (s)
Hilal Yuksel, 2017, Turkey [35]	To determine whether breathing exercises for pregnant women during the second stage of labour have beneficial effects on maternal pain, duration of labour, and the first-minute, APGAR scores, RCT 250 (IG-125, CG-125)	Nulliparous pregnant women in the second stage of labour with a gestational age ranging between 37 and 42 weeks. Those using analgesics or anaesthetics, and those with clinical instability, psychiatric disorders and the inability to cooperate with breathing exercises were excluded.	The main components of breathing exercises during training were: First, fill your stomach and then your lungs with air while breathing in. Feel the expansion in the stomach. Make sure the muscles from your stomach to your knee are relaxed, as if you are urinating while breathing out. When there is pain, perform deep abdominal breathing exercises, and take a deep breath in and hold as much as you can. Try to push the baby downward. You can do it by holding your breath or breathing out quite slowly from your mouth. The most important point in this stage is that you should not fill up the stomach with air, and you should push downward to deliver the baby. You should continue the pushing until the pain is relieved.	Standard care	The perception of the pain and duration of the second stage of labour was lower in the interventional group (369.6 ± 92) seconds as compared to the control group (440.7 ± 142.5) seconds, (*P* < 0.001). The mean first-minute APGAR scores were higher in the intervention group (8.84 ± 0.50) as compared to the control group (8.73 ± 0.89), (*P* > 0.05).
Lisa Kane Low, 2013, USA [33]	To test the effect of spontaneous pushing (either with or without prenatal perineal massage) compared with directed pushing on incontinence outcomes in women evaluated one year after their first birth. RCT (Solomon four group design), 249 (G1-39, G2-34, G3-32, G4-40).	Participants were pregnant women who are at least 18 years of age, no history of genitourinary pathology, continent during first 20 weeks of pregnancy by self-report, and continent at 20 weeks gestation by negative standing stress test. Women with demonstrable stress incontinence were excluded.	Group 1: directed pushing, or coached pushing using a closed glottis Valsalva maneuver. Group 2: prenatal perineal massage initiated in the third trimester with a standardized training regarding its use and then directed pushing during second-stage labour. Group 4: combination of group 2 and 3 treatment, with spontaneous pushing plus perineal massage.	Group 3: spontaneous pushing, with instruction provided prenatally via a standardized training video. This method included instructing the woman to follow her bodily sensations and push as she felt the urge.	The duration of second stage of labour in minutes was lower in Group 4 (spontaneous pushing and perineal massage) as compared to other groups. However, the results were not statistically significant (G1-131.12 ± 91.08, G2-130.28 ± 126.67, G3-151.69 ± 133.26, G4-104.19 ± 88.08, *P* = 0.47). Spontaneous pushing did not reduce the incidence of postpartum incontinence experienced by women one year after their first birth.
Sevil Cicek, 2017, Turkey [36]	To assess the effects of breathing techniques training on anxiety levels of pregnant women and the duration of labour. RCT, 70 (IG-35, CG-35).	Participants consisted of nulliparous women aged 18-35 years, 38-42 weeks pregnant with a single healthy foetus in vertex position, and expected to have spontaneous vaginal delivery without any pregnancy complications and in the early latent phase of labour (0-1 cm).	The four stages of breathing in the Lamaze breathing model were taught. In the latent phase (0-4cm) slowly inhale through nose and exhale through mouth. Then inhale through nose to a count of five seconds and exhale through mouth with the same slow way in five seconds. In the active phase (4-8 cm) breathe without using abdominal muscles with upper lungs. Accelerate and lighten your breathing as the contraction increases in intensity. Breathe in and out rapidly through mouth. In the transition phase (8-10 cm) breathe in and out through mouth. Blowing should be rapid and shallow.	Routine care	There were significant differences between the two groups regarding the mean State Anxiety Inventory (SAI) at late active phase of labour (*P* < 0.001). There were significant differences between the two groups regarding and the mean duration of first stage of labour (*P* < 0.001). The X ± SD of the duration of second stage of labour was (19.11 ± 12.49) minutes and (24.48 ± 16.32) minutes in the experimental and control group respectively with (*P* = 0.135).
Steven L Bloom, 2006, USA [34]	To compare obstetrical outcomes associated with coached vs uncoached pushing during the second stage of labour. RCT, 20 (IG-163, CG-157).	Participants were those with a singleton foetus in cephalic presentation and regular uterine contractions with cervical dilatation of at least four cm. Women with a prior history of urinary incontinence, anal incontinence, pelvic organ prolapse, any known complication of pregnancy, or an estimated foetal weight greater than 4000 g were excluded.	Step 1: head of bed up 30 degrees. Step 2: position patient, as she desires, on her back or either side. Step 3: coach patient to pull back on both knees and tuck her chin while the provider or partner supports the legs. Step 4: coach the patient to take a deep breath and hold during the peak of a contraction then bear down and push for 10 seconds; repeat this as long as the contraction continues.	Step 1: head of bed up 30 degrees. Step 2: position patient, as she desires, on her back or either side. Step 3: the patient should be told simply to “do what comes naturally” or whatever the patient feels the urge to do while in bed.	The second stage of labour was abbreviated by approximately 13 minutes in coached women (IG-46.3 ± 41.5, CG-59.1 ± 49.1, *P* = 0.014). There were no other clinically significant immediate maternal or neonatal outcomes between the two groups.
A Boaviagem, 2017, Brazil [37]	To assess the efficacy of the breathing patterns during the active phase of the first stage of labour for maternal anxiety. RCT, 140 (IG-67, CG-73).	Participants were parturient in active labour, aged 12-40 years, with gestational age between 37 and 41 weeks. Those with multiple pregnancies, pregnancy with a dead foetus, analgesic use, clinical instability and psychiatric disorders were excluded from the study.	The patient was instructed to inhale slowly, count from one to five and breathe out gradually, counting from five to one. For the breathing pattern with post exhalation pause, the patient was instructed to take a deep breath and increase the post-exhalation pause (one-two seconds). With respect to expiratory deceleration, the patient was instructed to take an extended exhalation, propelling the lips forward (pursed lip breathing).	Routine care	There was no difference between groups two hours after the first evaluation regarding to anxiety (MD = 0.3 (95% CI = -4.2, 4.8)), pain (MD = 0.0 (95% CI = -0.8, 0.7)), fatigue (MD = -0.5 (95% CI = -1.4, 2.5)) and maternal satisfaction (MD = 0.9 (95% CI = -0.1, 2.0). The labour duration measured in hours, found that the duration in intervention group was 7.73 ± 3.22 and control group was 8.02 ± 2.52, showing a significant difference, with a mean of 0.28 (95% CI = 1.32-0.75).
Sushmitha R Karkada, 2022, India [38]	To explore the impact of antepartum breathing exercises on maternal outcomes of labour among primigravida women. RCT, 261 (IG-138, CG-123).	Women were eligible to enter the trial if they had a singleton pregnancy with a cephalic presentation, had low risk (no pre-existing medical complications or existing obstetric complications), and were first-time childbearing women (primigravida). Women were excluded from entering the trial if they had pre-identified risk factors like eclampsia, preterm labour, placenta previa, multiple gestation, malpresentation and malposition or had been previously randomized to the trial.	Five breathing patterns were introduced namely- cleansing breathing for relaxation, slow-paced breathing, modified-paced breathing and patterned-paced breathing. Breathing patterns were demonstrated by the investigator to the women on a one-to-one basis. Women were asked to repeat these breathing patterns immediately after teaching and were advised to practice them twice daily for 15 minutes. Instructions were given to continue during the active phase of the first stage of labour under the supervision of labour room nurses.	The women randomized to the standard care group received health talk on antepartum care and services according to local health care provision.	A total of 98 (70%) primigravida women who practised antepartum breathing exercises had spontaneous onset of labour. The odds of spontaneous onset of labour after randomization in the intervention group was 2.192 times more when compared to standard care at a (95% CI = 1.31-3.36, *P* < 0.001). The requirement for augmentation of labour was minimal and there was a reduction in the rate of caesarean deliveries (*P <* 0.05) based on the χ^2^ test. A statistically and clinically significant difference was found in the mean duration of labour (in hours) between intervention 5.5127 (SD = 1.998) hours and standard care group 7.238 ± 3.678 h, resulting in a mean of 132 minutes, *P* < 0.001.
J I Schaffer, 2005, USA [30]	To determine effects of coached vs uncoached maternal pushing during the second stage of labour on postpartum pelvic floor structure and function, RCT, 128 (IG-67, CG-61).	Nulliparous women in spontaneous active labour with uncomplicated pregnancies between 36 and 41 weeks gestation, has regular uterine contractions, cervical dilatation of at least four cm, and foetuses in cephalic presentation. Women with a previous history of urinary incontinence, anal incontinence, pelvic organ prolapse, any known complication of pregnancy, or an estimated foetal weight greater than 4000 g were excluded.	Step 1: head of bed up 30 degrees. Step 2: position patient, as she desires, on her back or either side. Step 3: coach patient to pull back on both knees and tuck her chin while the provider or partner supports the legs. Step 4: coach the patient to take a deep breath and hold during the peak of a contraction then bear down and push for 10 seconds; repeat this as long as the contraction continues.	Step 1: head of bed up 30 degrees. Step 2: position patient, as she desires, on her back or either side. Step 3: the patient should be told simply to “do what comes naturally” or whatever the patient feels the urge to do while in bed.	Duration of second stage of labour was shorter in coached women in comparison to the uncoached women IG-3/67, CG-5/61, *P* = 0.385). No significant differences were found in prolonged second stage of labour, episiotomy, anal sphincter laceration, macrosomia, epidural, forceps, or oxytocin use.
Kirandeep Kaur, 2013, India [39]	To assess the effect of video on breathing exercises during labour on pain perception and duration of labour among the primigravida. Quasi experimental design, 40 (IG-20, CG-20).	Forty primigravida who were admitted in labour room were selected by purposive sampling technique. Mothers with respiratory diseases such as asthma, tuberculosis abdominal/uterine surgery were excluded from the study.	A video film of Hindi version (duration 10 minutes) which was developed with the storyline on breathing exercises during first stage of labour (slow breathing, fast breathing, pant-pant blow) and for second stage of labour (breathing exercises during childbirth) was shown prior to the onset of labour and re-demonstrations was obtained.	Routine care	The practice of breathing exercises during labour help to reduce pain perception and duration of first and second stage of labour. Pain perception at the latent, early and late active phases of first stage of labour showed statistical significant difference among experimental and control group (*P* < 0.01). Statistical significant difference (*P* < 0.01) was also observed in the duration of first stage of labour with mean duration (eight hours 48 minutes) in experimental group as compared to control group (nine hours 48 minutes). The mean duration of second stage of labour was also significantly less (*P* < 0.01) i.e. 24 minutes in experimental group as compared to 32 minutes in control group.
Tyseer Marzouk, 2019, Egypt [40]	To evaluate effectiveness of breathing exercise on reducing pain perception and state anxiety among primi parturients. Quasi-experimental design, 118 (IG-59, CG-59).	Participants were primi parturient in active phase of labour (i.e. ≥4 cm cervical dilation), aged between 20 and 35 years, at gestation weeks of 37 or beyond, did not receive analgesic or anaesthetic medication during the previous six hours, and not known to have a pre-existing respiratory disorders that may impair applying the breathing exercise.	Sit comfortably in leather armchairs during the exercise time. Keep one hand on the chest and the other on the abdomen at the umbilicus level. Gradually inspire air for four seconds; while nose in supine state. Slowly expire the inhaled air within six seconds through pursed lips; producing “Hoo” sound. Repeat step two and three during each contraction. During periods of rest, participants were taught to take breaths as in normal state.	Routine care	There was significant decline in women's perception of pain and state-anxiety in intervention group compared to control group (4.6 ± 2.0 vs. 5.9 ± 1.8 and 60.0 ± 7.8 vs. 64.3 ± 8.8) respectively. Intervention group subjects perceived lower pain and experienced lower state-anxiety compared to those of the control group after two hours (4.4 ± 2.1 vs. 5.8 ± 1.7 and 57.1 ± 7.8 vs. 63.8 ± 8.8) and after four hours (3.6 ± 1.4 vs. 5.7 ± 1.6 and 53.7 ± 7.8 vs. 63.3 ± 8.9 respectively. The parturient women who performed the breathing exercise had significantly shorter duration of the active phase of first stage of labour than those in the control group (5.9 ± 0.8 vs. 7.9 ± 0.8) hours, *P* < 0.001, while the duration of 2^nd^ stage of labour did not differ significantly between the two groups (49.5 ± 4.5 vs. 50.4 ± 1.7) minutes, *P* = 0.160.

IG – intervention group, CG – control group, RCT – randomized controlled trial, G1 – group-1, G2 – group-2, G3 – group-3, G4 – group-4, MD – mean difference, CI – confidence interval

**Figure 3:**
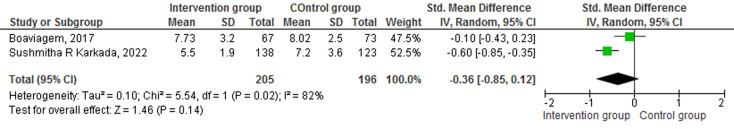
Effect of breathing exercise on the duration of labour.

The continuous variables of the trials of the existing review were analysed, and a meta-analysis is reported using mean and standard deviation among the intervention and control groups. The intervention group received breathing exercises as an intervention, while the control group received standard care. Three trials assessed the effectiveness of breathing exercises on the duration of labour [37,38]. The results of the efficacy of breathing exercise on the duration of labour were pooled for analysis and found that there was not a statistically significant difference between the intervention and control group SMD = -0.36 (95% CI = -0.85, -0.12), *P* = 0.14, *Z* = 1.46 (**Figure 3**).

**Figure 4:**
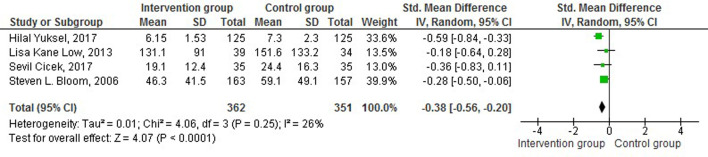
Effect of breathing exercise on the duration of second stage of labour.

Four trials reported the duration of the second stage of labour. The intervention group received breathing exercises as an intervention, while the control group received standard care. The Forest plot depicts that the breathing exercise shortened the duration of the second stage of labour, and statistically significant difference between the intervention and control group SMD = -0.38 (CI 95% = -0.56, -0.20), *P* < 0.0001, *Z* = 4.07 [33,34,35,36] (**Figure 4**).

**Figure 5:**
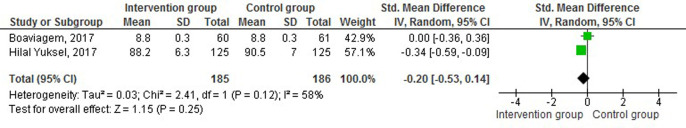
Effect of breathing exercise on the duration of pain.

The secondary outcomes included in this study were anxiety, duration of pain, APGAR scores, episiotomy, and mode of delivery. Six trials assessed the effect of breathing exercises on secondary outcomes. Trials did not show a statistically significant effect of breathing exercise on APGAR score at one [35] and five minutes [34,37]. One trial showed a statistically significant effect of breathing exercise on pain [35] and anxiety [36], and one trial reported that there is not a statistically significant effect of breathing exercise on anxiety [37] and pain [37]. The results on the effectiveness of breathing exercise on the duration of pain were pooled for analysis and found that there is no statistically significant effect of breathing exercise on the duration of pain between the intervention and control group SMD = -0.20 (95% CI = -0.53, 0.14), *Z* = 1.15, *P* = 0.25 [35,37] (**Figure 5**).

**Figure 6:**
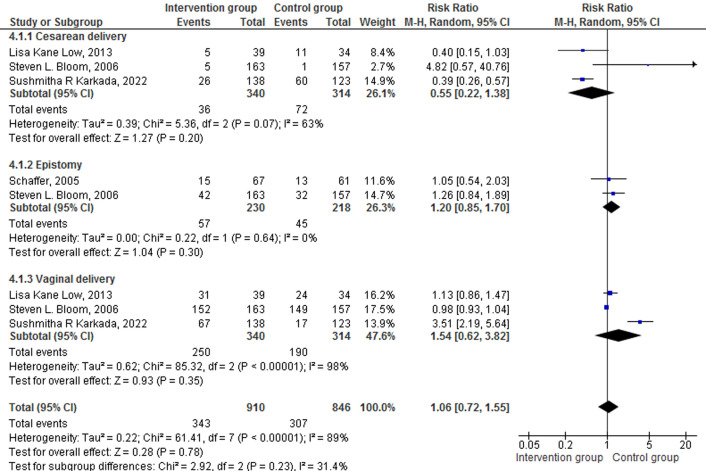
Effect of breathing exercise on caesarean delivery, vaginal delivery, and episiotomy.

The secondary outcomes of dichotomous variables such as mode of delivery (caesarean delivery, vaginal delivery), and episiotomy were analysed and reported as RR using random effect model. Three studies reported the efficacy of breathing exercises on the mode of delivery among pregnancy in intervention and control groups [33,34,38]. Participants who practised breathing exercises had a 45% lower risk for caesarean delivery compared to participants who did not practice breathing exercises RR = 0.55 (95% CI = 0.22-1.38), *P* = 0.20, *Z* = 1.27. Similarly, participants who practised breathing exercises had 1.54 times more chances of having vaginal delivery as compared to the control group RR = 1.54 (95% CI = 0.62-3.82), *P* = 0.35, *Z* = 0.93. Two trials reported the need for episiotomy during the second stage of labour [30,34]. Participants who practised breathing exercises had 1.20 times more chance of having episiotomy as compared to the control group RR = 1.20 (95% CI = 0.85-1.70), *P* = 0.30, *Z* = 1.04 (**Figure 6**).

## DISCUSSION

Our systematic review and meta-analysis found that breathing exercise shortens the duration of the second stage of labour. In the narrative synthesis, seven trials and two quasi-experimental studies concluded that breathing exercise shortens the total duration of labour and the second stage of labour. None of the included studies reported any harmful effects of breathing exercise intervention.

Breathing exercise during labour involves deep inhalation and exhalation. Performing this exercise helps in the mobilization of the muscles of the pelvic floor, and the muscles of the abdomen are actively contracted and oxygenated [41]. Continuous support during labour significantly enhances the labour physiology and the mother’s feelings of control and competence, decreasing dependency on medical interventions [42]. Effective and safe strategies that have the potential to reduce the discomfort of women in labour are important.

Different types of CAM have proved efficacious in reducing labour pain [43], physiological and psychological stress [44], anxiety [45], and tension headache in pregnancy [46]. There is also evidence available that women who are physically active during pregnancy have a shorter duration of active labour [47,48], lower number of perineal tears [49], reduction in CS rates [50] and appropriate maternal and foetal weight gain [50].

The duration of labour is a detrimental factor in the pregnancy outcome and maternal and neonatal complications. In an infant, a prolonged duration of labour could result in choking, neuro-physiological complications, and death. Furthermore, women with a longer duration of labour are vulnerable to postpartum haemorrhage, psychological distress and fatigue [3,51,52]. Negative birth experience during the first labour is associated with a subsequent wish for CS in the next labour or deciding not to have any more children, and prolonged labour is one of the major factors contributing to such a request [15,53,54]. Globally, it is predicted that the CS rate will be nearing 30%, with 38 million CS performed in the year 2030 [55]. Women undergoing CS have a higher incidence of subsequent miscarriage, placenta praevia and accreta, and children born by CS have a higher incidence of asthma and obesity than children born vaginally [56]. However, breathing exercise during the first stage of labour was not found to be effective in increasing maternal satisfaction [37]. This may be attributed to the fact that maternal satisfaction is influenced by various factors, namely planned childbirth, decreased wait time, support received from the health care professionals, birth room infrastructure, patient-caregiver relation, and their involvement in decision-making [57-60].

This meta-analysis provides some evidence regarding the effectiveness of breathing exercise on the total duration of the labour and the second stage of labour. Although breathing exercise alone may not be adequate to improve the positive health status, we contemplate that the findings of this systematic review and meta-analysis are significant contribution to the scope of research on breathing exercise among women in labour. Midwives are in a unique position to administer breathing exercise intervention, as prolonged duration of labour has negative consequences on the present and future labour. However, lack of time, lack of knowledge, inadequate staffing, and patient unwillingness are barriers to the successful implementation of breathing exercise during labour.

The limitations of the reviewed articles were that the RCTs focused on low-risk pregnant women without any major complications, single-blinded study, and limited generalizability as the participants were recruited from a single centre. The review has limitation of language bias as the articles published in English language were only involved. Attributed to lesser number of studies included in the meta-analysis, publication bias was not assessed.

## CONCLUSIONS

Breathing exercise is an effective complementary preventive intervention in shortening the duration of the second stage of labour. With the adverse consequences associated with amniotomy, oxytocin and CS, complementary therapies could facilitate a favourable delivery experience.
